# Supplementary material for: Framework with cytoskeletal actin filaments forming insect footpad hairs inspires biomimetic adhesive device design
Source: Commun Biol. 2020 May 29;3:272. doi: 10.1038/s42003-020-0995-0 (PMC7260203; doi:10.1038/s42003-020-0995-0)
Supplement: Supplementary file 1 — Description of Additional Supplementary Files [file 42003_2020_995_MOESM1_ESM.pdf]

Description of additional supplementary items

SUPPLEMENTARY DATA FILE LEGEND

Supplementary Data 1.
The file contains the raw data and p values underlying each graph of Figures used in this study.

SUPPLEMENTARY MOVIE LEGENDS

Supplementary Movie 1. Time-lapse images of a developing footpad in *Drosophila*
Time-lapse images monitoring the developmental changes occurring during the formation of a footpad between 23 h and 60 h APF in a pupa from a fly of *spa>mCD8::GFP, nucDsRed*. The footpad-forming cells and their nuclei were visualized with mCD8::GFP (green) and DsRed (red) fluorescence, respectively, under the control of *svp-GAL4*. Images were captured every 20 min. Lateral view. Distal is to the right.

Supplementary Movie 2 Three-dimensional reconstitution image of MARCM labeled cells forming a hair in *Drosophila*.
The movie was constructed from the digital sectioning of the image in Supplementary Figure 3. Note that a single cell extends a cellular process, forming the shaft of a single hair.

Supplementary Movie 3 Time-lapse images of a developing footpad in an *Act5C* knockdown fly
Time-lapse images monitoring the developmental changes occurring during the formation of a footpad between 24 h and 42 h APF in a pupa from a fly of *svp> mCD8::GFP,Act5C RNAi, elav-Gal80*. The footpad-forming cells were visualized with mCD8::GFP (green) fluorescence under the control of *svp-GAL4*. Images were captured every 20 min. Lateral view. Distal is to the right.

Supplementary Movie 4 Climbing assay of wild-type flies with normal footpads in *Drosophila*

The flies used were of the genotypes +*:* + (*CS* strain). Images of climbing flies on a Pasteur pipette were captured for over 1 min. Double-speed.

Supplementary Movie 5 Climbing assay of *Act5C* knockdown flies with malformed footpads in *Drosophila*

The flies used were of the *svp>Act5C RNAi, elav-Gal80.* Images of climbing flies on a Pasteur pipette were captured for over 1 min. Double-speed.
