# Supplementary material for: Framework with cytoskeletal actin filaments forming insect footpad hairs inspires biomimetic adhesive device design
Source: Commun Biol. 2020 May 29;3:272. doi: 10.1038/s42003-020-0995-0 (PMC7260203; doi:10.1038/s42003-020-0995-0)
Supplement: Supplementary file 2 — Supplementary Information [file 42003_2020_995_MOESM2_ESM.pdf]

Supplementary Figures

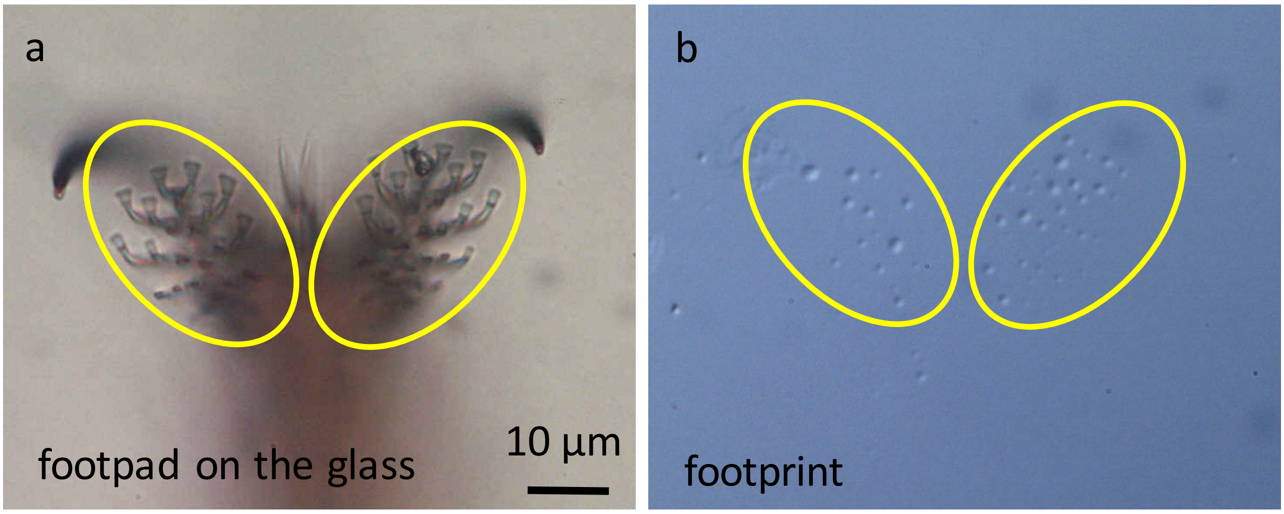

Supplementary Figure 1 Footpad of a walking fly and its footprint

(a) Photograph of a wild-type *Canton-Special* (*CS*) fly walking along the surface of a glass coverslip. Many hair tips are attached to the glass surface. (b) The residual footprint of the fly observed with a Nomarski microscope (Olympus, AX70). Liquid droplets are seen on the glass in a similar pattern to that of the hair on the footpad. Scale: 10 µm.

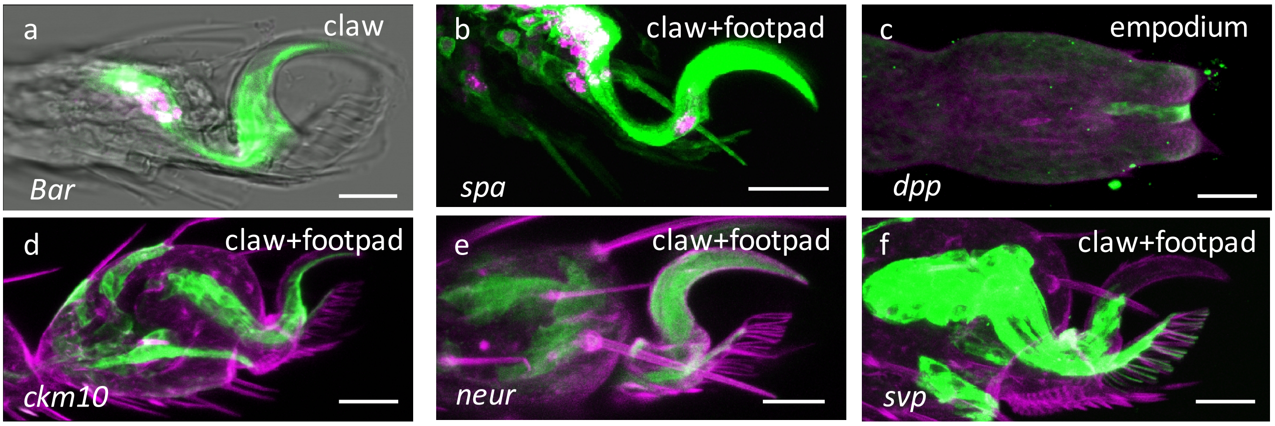

Supplementary Figure 2 Screening for suitable GAL4 lines for labeling footpad cells with GFP

(a) *Bar>mCD8::GFP, nucDsRed*. Most of the claw cells are labeled with GFP. (b) *spa>mCD8::GFP, nucDsRed*. Most of the claw cells and some of the footpad cells are labeled GFP. (c) *dpp>mCD8::GFP*. Empodium cells are labeled with GFP. (d) *ckm10>mCD8::GFP*. Most of the ventral claw cells and some of the footpad cells are labeled with GFP. (e) *neur>mCD8::GFP*. Most claw cells and some footpad cells are labeled with GFP. (f) *svp>mCD8::GFP*. Some of the ventral claw cells and most of the footpad cells are labeled with GFP. Pretarsal cells are outlined by fluorescent phalloidin staining (magenta) (c-f). Scale: 20 µm.

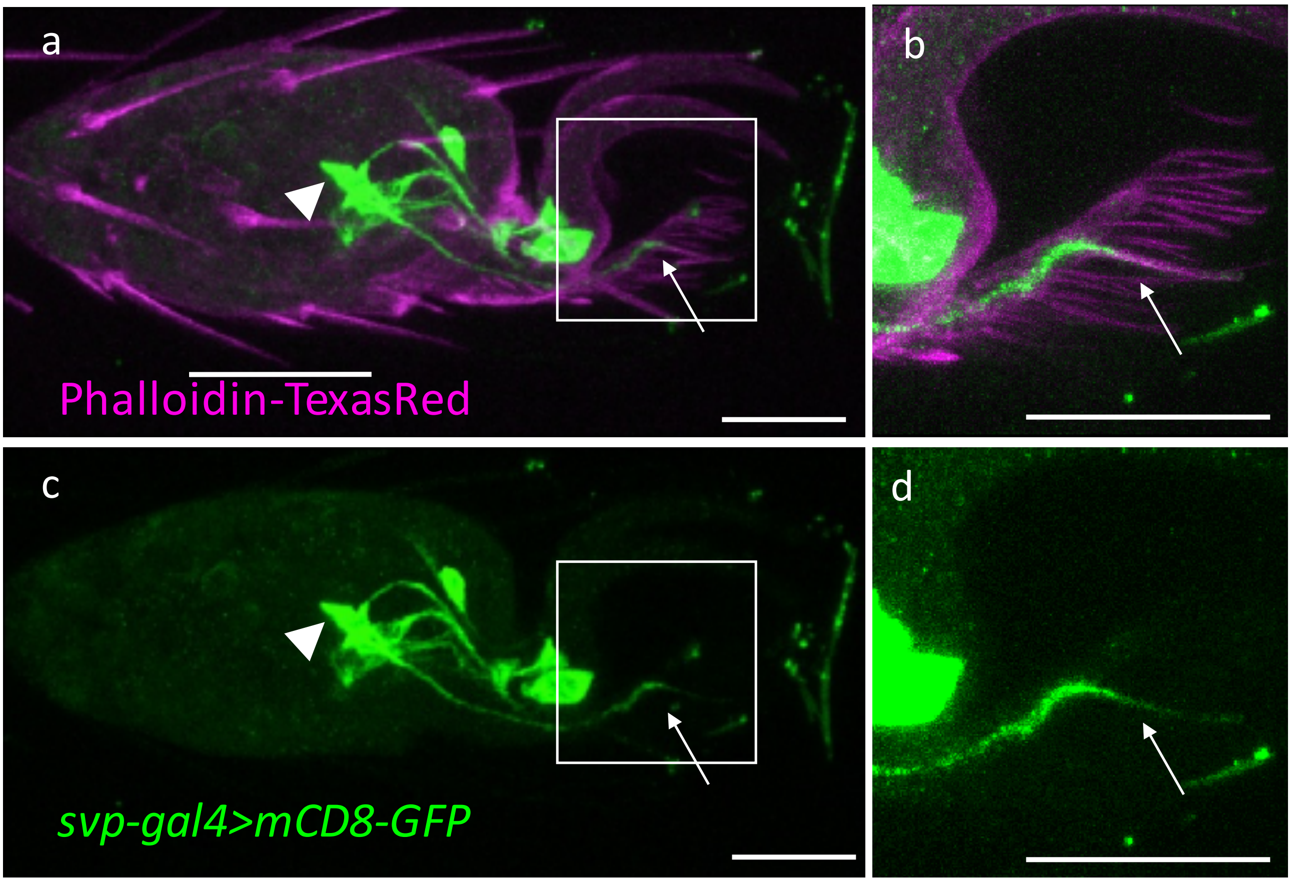

Supplementary Figure 3 MARCM labeling of cells forming a hair in *Drosophila*

(a-d) Tarsal and pretarsal regions labeled with fluorescent phalloidin (magenta) in a pupa of *y hs-flp; FRTG13, UAS-mCD8::GFP/ FRTG13, TubP-Gal80;svp-Gal4*/+ at 42 h APF. A limited number of *svp*-expressing cells were labeled with GFP (green) by the MARCM method. A single cell (arrowhead in a, c) extends a cellular process, forming the shaft of a single hair (arrow in b, d). The boxed regions in (a) and (c) are enlarged in (b) and (d), respectively, highlighting the tips of processes in hair cells. Scale: 20 µm.

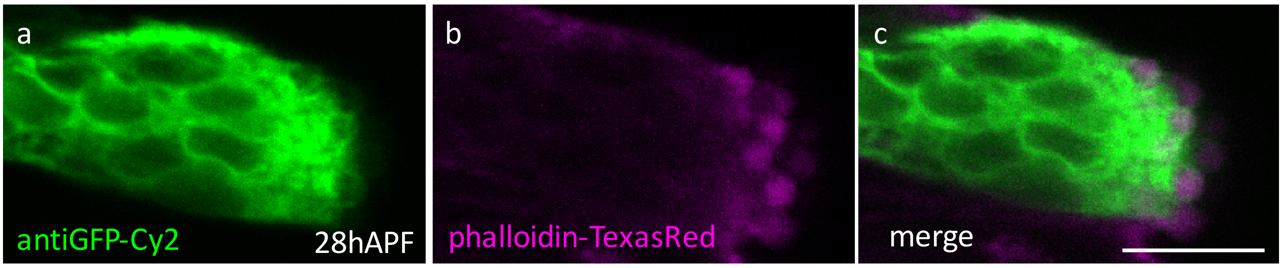

Supplementary Figure 4 Accumulation of cytoskeletal actin at the tips of footpad's hairs

(a-c) TexasRed-conjugated phalloidin staining of the pretarsus at 28 h APF in an *FRTG13, UAS-mCD8::GFP;svp-Gal4/TM6b* fly. (a) mCD8::GFP (green). (b) TexasRed staining (magenta). (c) Merged image. Scale: 20 µm.

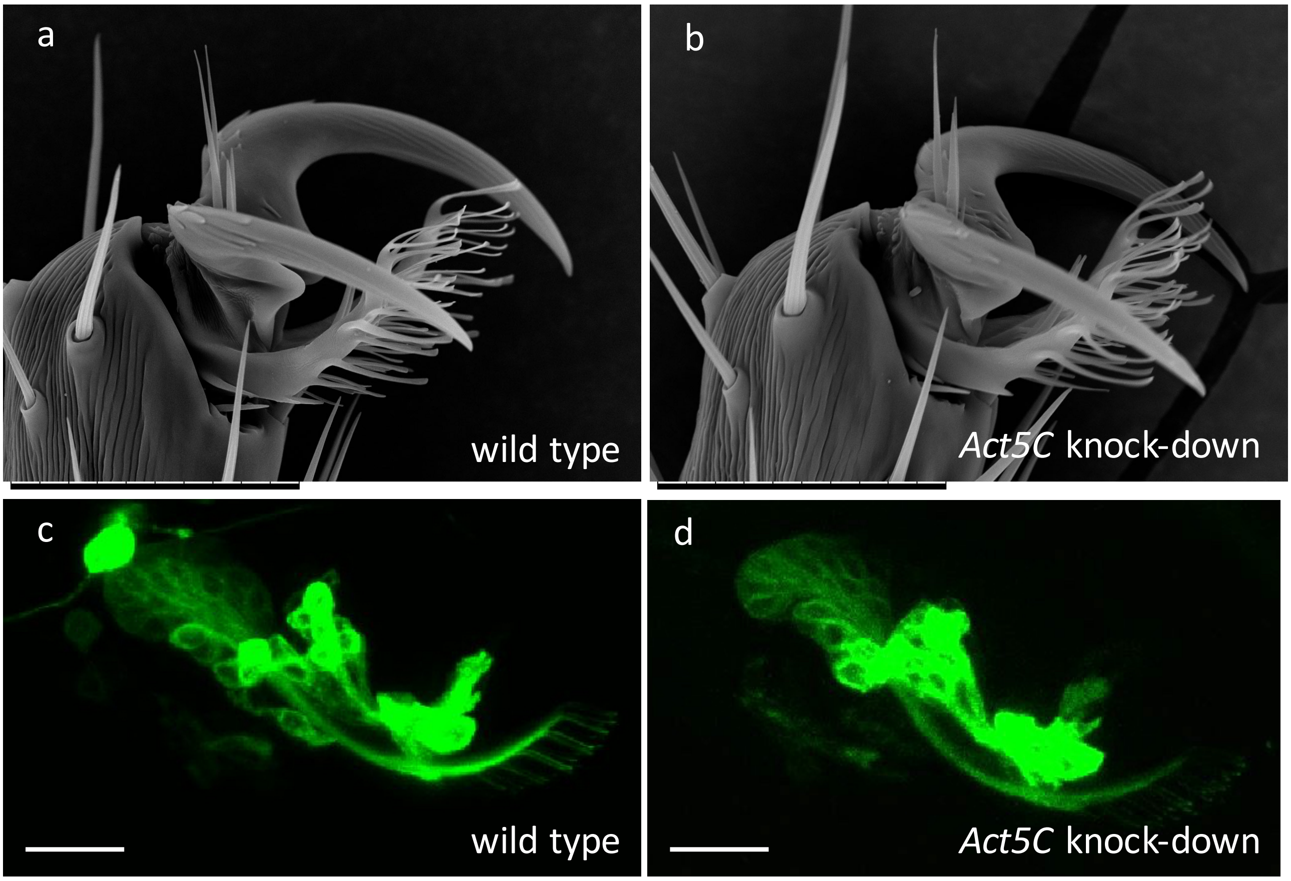

Supplementary Figure 5 Morphology of footpad and its formation process in wild-type and *Act5C* knockdown flies

(a, b) Adult footpad in wild-type (CS) and *Act5C* knockdown fly (*svp>Act5C RNAi, elav-Gal80*), respectively. Scale: 30 µm. (c, d) Footpad-forming cells labeled with mCD8-GFP at 42 h APF in wild-type (*svp>mCD8::GFP*) and *Act5C* knockdown fly (*svp>mCD8::GFP, Act5C RNAi, elav-Gal80*), respectively. The retraction of hair cells occurred both in wild-type and *Act5C* knockdown flies. Scale: 20 µm.

Supplementary Table

Supplementary Table 1 Detailed genotypes and the abbreviations of all strains used in the study

Fig. 1		
a, b, c	*CS*	*+; +*
d-g'	*svp>Act5C::GFP, nucDsRed*	*UAS-Act5C::GFP/+; svp-GAL4/ UAS-nucDsRed*
Fig. 2		
a, b	*CS*	*+; +*
c-c'''	*svp>mCD8::GFP*	*FRTG13, UAS-mCD8::GFP; svp-GAL4/TM6b*
d,e,f	*svp>Act5C RNAi, TubP-GAL80*^{TS}	*UAS-Act5C RNAi/ TubP-GAL80*^{TS}*; svp-GAL4/+*
	svp>Act5C RNAi, elav-Gal80	*UAS-Act5C RNAi/ +; svp-Gal4/elav-Gal80*
	CS	*+; +*
Fig. 3		
a, b	*CS*	*+; +*
	+/ +; svp-GAL4/+	*+/ +; svp-GAL4/+*
	UAS-Act5C RNAi/+; elav-GAL80/+	*UAS-Act5C RNAi/ +; elav-GAL80/+*
	svp>Act5C RNAi, elav-Gal80	*UAS-Act5C RNAi/ +; svp-Gal4/elav-Gal80*
Supplementary Fig. 1		
	CS	*+; +*
Supplementary Fig. 2		
a	*Bar>mCD8::GFP, nucDsRed*	*v*^{36f}*, BN-GAL4/Y; UAS-mCD8::GFP/+; UAS-nucDsRed /ry*^{506}
b	*spa>mCD8::GFP, nucDsRed*	*spa-GAL4/Y; UAS-mCD8::GFP/+; UAS-nucDsRed/+*
c	*dpp>mCD8::GFP*	*w; UAS-mCD8::GFP/+; blk-GAL4, UAS-GFPnls/+*
d	*ckm10>mCD8::GFP*	*v*^{36f}*, BN-GAL4/Y; UAS-mCD8::GFP/+; +/ry*^{506}
e	*neur>mCD8::GFP*	*UAS-mCD8::GFP/+; Gal4-A101 Kg*^{V}*/+*
f	*svp>mCD8::GFP*	*UAS-mCD8::GFP/+; svp*^{NP5606}*-Gal4/+*
Supplementary Fig. 3		*FRTG13, UAS-mCD8::GFP/ FRTG13, TubP-Gal80; svp-Gal4/+*
Supplementary Fig. 4		
	svp>mCD8::GFP	*FRTG13, UAS-mCD8::GFP; svp-Gal4/TM6b*
Supplementary Fig. 5		
a	*CS*	*+; +*
b	*svp>Act5C RNAi, elav-Gal80*	*UAS-Act5C RNAi/ +; svp-Gal4/elav-Gal80*
c	*svp>mCD8::GFP*	*FRTG13, UAS-mCD8::GFP; svp-GAL4/TM6b*
d	*svp>mCD8::GFP, Act5C RNAi, elav-Gal80*	*FRTG13, UAS-mCD8::GFP/UAS-Act5C RNAi; svp-Gal4/elav-Gal80*
Supplementary Movie 1		
	svp>mCD8::GFP, nucDsRed	*FRTG13, UAS-mCD8::GFP; svp-Gal4/UAS-nucDsRed*
Supplementary Movie 2		*FRTG13, UAS-mCD8::GFP/ FRTG13, TubP-Gal80; svp-Gal4/+*
Supplementary Movie 3		
	svp>mCD8::GFP, Act5C RNAi, elav-Gal80	*FRTG13, UAS-mCD8::GFP/UAS-Act5C RNAi; svp-Gal4/elav-Gal80*
Supplementary Movie 4		
	CS	*+; +*
Supplementary Movie 5		
	svp>Act5C RNAi, elav-Gal80	*UAS-Act5C RNAi/ +;svp-Gal4/elav-Gal80*
